# Olecranon Bursitis Secondary to Trauma

**DOI:** 10.7759/cureus.27306

**Published:** 2022-07-26

**Authors:** Shalini Subramanian, Trilok G Stead, Rohan K Mangal, Vashun Rodriguez, Latha Ganti

**Affiliations:** 1 Biology, University of South Florida, Tampa, USA; 2 Biology, Trinity Preparatory School, Winter Park, USA; 3 Medicine, University of Miami Miller School of Medicine, Miami, USA; 4 Emergency Medicine, Lakeland Regional Health, Lakeland, USA; 5 Emergency Medicine, HCA Florida Ocala Hospital, Ocala, USA; 6 Emergency Medicine, Envision Physician Services, Plantation, USA; 7 Emergency Medicine, University of Central Florida College of Medicine, Orlando, USA

**Keywords:** tissue, disease, aseptic, skin, olecranon bursitis

## Abstract

The authors present a case of traumatic olecranon bursitis, initially presumed to be cellulitis. The clinical presentation, diagnosis, and management are discussed.

## Introduction

Bursae are fluid-containing sacs that reduce friction between tissue layers during movement [1]. Repetitive friction of the bursa tissue can result in bursitis [2]. A common presentation of bursitis occurs at the olecranon due to its location and minimal vascularity making it more subject to trauma [3]. Two-thirds of olecranon bursitis cases are aseptic, the remaining of which are considered septic or infected [4,5]. The incidence of olecranon bursitis in a study conducted in the military population is estimated to be 0.01% and increases to 0.1% in inpatient populations [6,7]. The incidence rate may be underrepresented since many cases of olecranon bursitis can resolve spontaneously or with conservative treatment [3,8]. Superficial olecranon bursitis often results from leaning on elbows with pressure, causing acute trauma and swelling. Repetitive trauma can cause open wounds that are susceptible to bacterial invasion, thereby leading to septic bursitis [7]. One study reported that 19.8% of olecranon bursitis cases resulted in a complication such as infection [9]. While a variety of microbes have been found to produce septic olecranon bursitis, the most common causative agent is *Staphylococcus aureus* [7]. Middle-aged men are at a higher risk of being affected, especially if they participate in combat sports or conduct heavy manual labor [6]. Additionally, individuals on chronic dialysis may experience olecranon bursitis, validating the mechanism of microtrauma from resting on the elbow that occurs during dialysis procedures [10]. Olecranon bursitis can also develop as a result of lowered immunity and preexisting medical conditions such as rheumatoid arthritis, gout, and diabetes. While gout can cause various forms of bursitis, it tends to be a significant contributor to olecranon bursitis due to the settling of monosodium urate crystals in anatomical areas of low temperatures [7].

## Case presentation

**Figure 1:**
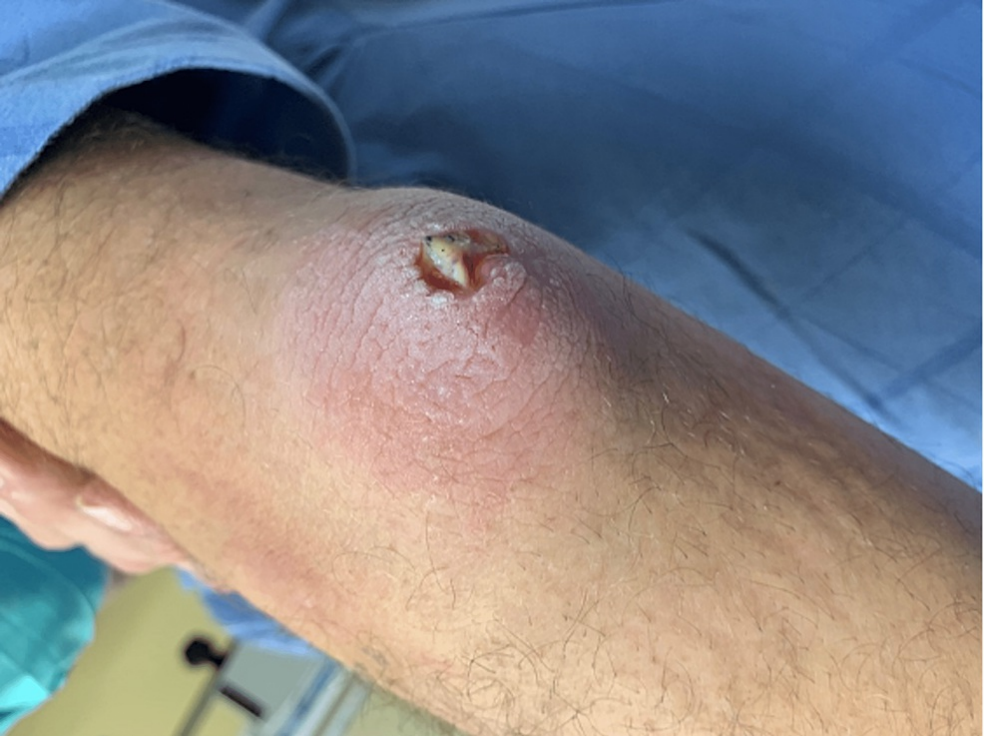
Clinical photograph of the patient’s arm.

The patient is a 50-year-old gentleman who arrived at the emergency department (ED) due to the swelling of his left elbow. He recalled that he hit his elbow on the side of the swimming pool while jumping in, followed by immediate swelling. His medical history was positive for hypertension and smoking. His surgical history included a prior knee surgery. The patient took over-the-counter analgesics for mild pain. He noticed the swelling persisted, and there was a scab over the elbow with a small surrounding area of redness. The patient’s primary care doctor prescribed cephalexin, presuming it to be cellulitis. The patient picked at the scab and opened it (Figure 1). 

**Figure 2:**
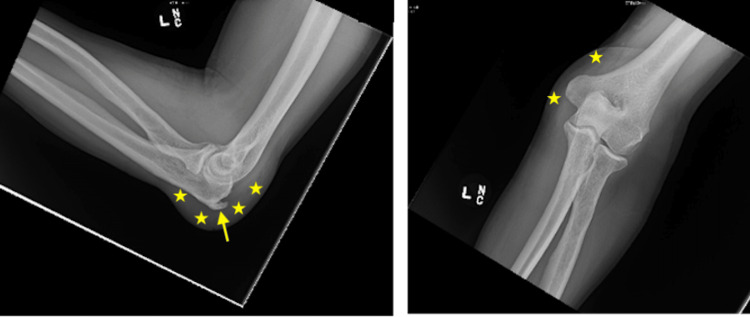
Plain radiographs of the left elbow demonstrating an olecranon spur (arrow) and inflammation of the fluid-filled bursa (stars).

While it did not bother him much, his family wanted him to get a second opinion, so he came to the ED. Vital signs revealed a temperature of 98.6 °F, a pulse of 98 beats per minute, respiratory rate of 16 breaths per minute, a blood pressure of 155/91 mmHg, and oxygen saturation of 98% on room air. Physical examination revealed mild edema and erythema about the elbow but no calor. Plain radiographs (Figure 2) did not show any evidence of acute fracture, dislocation, or suspicious osseous lesion.

An olecranon spur was noted as was a 1.8 cm soft tissue thickening at the posterior elbow, consistent with olecranon bursitis. The patient was discharged home with a cushioned elbow bandage and instructions not to pick at the wound.

## Discussion

The clinical presentation of olecranon bursitis is characterized by an enlarged bursa, erythema, and pain on flexion [11,4,5]. Clinical examination alone may be inadequate to distinguish between superficial and septic bursitis. In this case, the diagnosis was also obscured by the open wound over the elbow secondary to trauma, leading the first physician to prescribe antibiotics. The recommendation for diagnosis of septic bursitis is through the analysis of the aspirated bursal fluid using ultrasonography. However, the procedure of aspiration can pose a high risk of sinus tract formation [5]. In our patient, given the lack of systemic symptoms, and this visit being several days post-injury, the likelihood of septic bursitis was very low. One study reported that a 2.2 °Celsius difference on the surface between the inflammation site and the nearby surface is 94% specific in distinguishing septic from aseptic forms [12]. Skin temperature, presence of fever, and elevated inflammatory markers can be used to help diagnose septic olecranon bursitis [5]. Anti-inflammatory medications, rest, ice, compression, and elevation (RICE) are considered the appropriate treatment for aseptic olecranon bursitis. The treatment of septic bursitis involves elbow orthosis, antibiotics, and compression bandaging [3,5]. In severe cases of septic bursitis, bursectomies, aspiration, or intrabursal injections may be considered, all of which may pose long-term effects such as poor wound healing, sinus formation, and possible tendon ruptures, respectively [3].

## Conclusions

Olecranon bursitis is a common cause of elbow swelling that can occur secondary to direct trauma, overuse, or infection. It is often mistaken for an infection, especially when there is an open wound secondary to trauma that overlies it. The mainstay of therapy involves rest and anti-inflammatory medications. The condition is benign and resolves in a few weeks.
